# The immunogenicity control axis: a multi-omics framework for integrating epigenetic, DNA damage response, and immune states in cancer

**DOI:** 10.3389/fgene.2026.1944709

**Published:** 2026-10-14

**Authors:** Manyata Srivastava, Shishir Singh, Diksha Ameta, Aesha Singh, Monisha Banerjee, Atar Singh Kushwah

**Affiliations:** 1 Department of Bioscience, Manipal University Jaipur, Jaipur, Rajasthan, India; 2 Division of Immunobiology, Cincinnati Children’s Hospital Medical Center, Cincinnati, OH, United States; 3 Department of Internal Medicine, Marshall University, Huntington, WV, United States; 4 Department of Zoology, University of Lucknow, Lucknow, India

**Keywords:** cervical cancer, DNA damage response, epigenetics, immunogenicity control axis, immunotherapy resistance, multi-omics integration, natural compounds, tumor immunogenicity

## Abstract

Immunotherapy has transformed cancer treatment, yet therapeutic responses remain heterogeneous and are incompletely explained by immune-centric biomarkers alone. We propose the Immunogenicity Control Axis (ICA), a testable multi-omics framework integrating three complementary dimensions of tumor state: epigenetic regulation, DNA damage response (DDR), and immune activity. The central hypothesis is that epigenetic permissiveness influences immune-relevant transcription and antigen presentation, while DDR-associated genomic processes contribute to antigenic variation; together, these tumor-intrinsic features interact with the immune state to shape tumor immune visibility. To make this hypothesis quantitatively testable, the ICA is operationalized through independently derived Epigenetic, DDR, and Immune scores that are standardized and integrated into a composite ICA Score. Using cervical cancer as an initial disease context, patient-derived TCGA-CESC analyses provide exploratory assessment of ICA-associated molecular states and prognostic relationships, while synthetic analyses are used separately to illustrate specific analytical procedures. The framework is further extended to perturbational analysis, in which CMap/LINCS transcriptional signatures and network information are used to prioritize candidate natural compounds predicted to oppose ICA-associated molecular states. These computational analyses are intended as hypothesis-generating operationalizations rather than biological or clinical validation. ICA therefore provides a falsifiable framework for testing whether coordinated epigenetic, DDR, and immune information captures tumor immunogenic variation beyond individual molecular dimensions and for prioritizing perturbational hypotheses for subsequent experimental validation.

## Introduction

Immune checkpoint blockade and related immunotherapeutic strategies have transformed cancer treatment; however, clinical responses remain heterogeneous and frequently limited to a subset of patients (Sharma and Allison, 2015; Topalian et al., 2015; Ribas and Wolchok, 2018). Many clinically used and widely studied immunotherapy biomarkers emphasize the lens of immune checkpoint signaling and immune cell infiltration (Topalian et al., 2015; Ribas and Wolchok, 2018). While informative, these models implicitly treat immune responsiveness as an intrinsic tumor property, largely independent of upstream regulatory constraints.

This reductionist view overlooks two fundamental determinants of tumor immunogenicity: epigenetic regulation and DNA damage response (DDR) fidelity. Epigenetic mechanisms, including histone acetylation and DNA methylation, directly regulate chromatin accessibility and thereby determine whether immune-relevant genes such as those governing antigen presentation and interferon signaling—are transcriptionally accessible (Roulois et al., 2015; Topper et al., 2017; Jones et al., 2019). In parallel, DDR pathways control the generation and structural architecture of genomic alterations, shaping both the quantity and immunological relevance of neoantigens available for immune recognition (Lord and Ashworth, 2017; Chan et al., 2019; Pilié et al., 2019; Kushwah et al., 2024a). Despite their central roles, these regulatory layers are typically studied in isolation and are not mechanistically integrated into current immunotherapy models.

This conceptual gap is particularly evident in cervical cancer, where viral oncogenesis, chromatin reprogramming, and DDR dysregulation coexist but fail to explain variability in immune response and therapeutic outcomes (The Cancer Genome Atlas Research Network, 2017). Human papillomavirus (HPV)-driven tumors exhibit persistent immune evasion despite the presence of viral antigens, suggesting that upstream regulatory constraints limit effective immune activation (Galluzzi et al., 2015; Spranger et al., 2015; Vinay et al., 2015; Kushwah et al., 2024b). Consequently, therapeutic strategies targeting individual components—immune checkpoints, epigenetic modifiers, or DDR pathways—often produce inconsistent and context-dependent responses (Herbst et al., 2014; Topalian et al., 2016; Sharma et al., 2017).

Unlike prevailing models that treat epigenetic regulation, DNA repair, and immune activation as parallel but independent processes, we propose a directionally integrated framework in which these layers form an interacting regulatory system hypothesized to shape tumor immunogenicity. Epigenetic state can constrain transcriptional accessibility of immune-relevant programs, whereas DDR-associated mutational processes can influence the genomic substrate from which candidate neoantigens arise (Alexandrov et al., 2013; McGranahan et al., 2016; Kushwah et al., 2024a). Beyond its mechanistic integration, the ICA is designed as an operational framework that enables multi-omics–based tumor stratification, computational prioritization of candidate natural compounds, and systematic hypothesis generation for precision immunotherapy.

We define this system as the Immunogenicity Control Axis (ICA), representing a mechanistically coupled network integrating epigenetic state, DNA repair pathway bias, and immune activation. Within the ICA hypothesis, tumor immunogenicity is conceptualized as a dynamic systems-level phenotype shaped by interacting molecular states. We further propose that selected natural compounds can be evaluated as candidate multi-target perturbators of ICA-associated molecular states. This perspective provides a testable and integrative foundation for interpreting immunotherapy variability and establishes a basis for multi-omics-driven precision immunomodulation.

The central proposition of this study is therefore biological: tumor immunogenicity is conceptualized as an emergent state shaped by coordinated interactions among epigenetic regulation, DNA damage response, and immune activity. The ICA Score, molecular-state analyses, prognostic benchmarking, and perturbational compound prioritization are presented as computational operationalizations and testable downstream applications of this central hypothesis, rather than as independent conceptual frameworks or validated clinical tools. This hierarchy is maintained throughout the manuscript to distinguish the core biological model from the analytical procedures used to interrogate it.

## Limitations of current models

### Immune-centric models

Immune-centric frameworks prioritize checkpoint pathways such as PD-1/PD-L1, immune-cell infiltration, and related measures that have demonstrated clinical relevance for immunotherapy stratification (Topalian et al., 2015; Van Allen et al., 2015; Cristescu et al., 2018; Ribas and Wolchok, 2018). However, comparable immune profiles do not necessarily produce equivalent therapeutic responses, because immune activity is conditioned by tumor-intrinsic determinants such as chromatin accessibility and genomic architecture (Herbst et al., 2014; Topalian et al., 2016; Sharma et al., 2017). Thus, immune-centric models characterize important downstream features of tumor–immune interaction but incompletely represent the upstream molecular constraints that determine whether immune-relevant programs can be effectively engaged.

### Epigenetic-centric models

Epigenetic frameworks establish chromatin accessibility and DNA methylation as major determinants of transcriptional state, including regulation of antigen-presentation and interferon-responsive programs (Feinberg et al., 2006; Seto and Yoshida, 2014; Jones et al., 2019). Their limitation for immunogenicity modeling is not failure to recognize immune regulation, but the absence of an explicit integration with the genomic processes that generate tumor-associated antigenic variation and with the immune context that determines its recognition. Epigenetic state therefore represents an important regulatory dimension of tumor immune visibility but is insufficient, in isolation, to account for the complete immunogenic phenotype (Roulois et al., 2015; Topper et al., 2017).

### DDR-centric models

DDR-focused frameworks explain genomic instability, mutational processes, and therapeutic vulnerabilities associated with defective or altered DNA (Jeggo et al., 2016; Lord and Ashworth, 2017; Pilié et al., 2019; Singh et al., 2025). DNA-repair defects can also influence tumor–immune interactions through mutation-derived antigenicity and other immunogenic consequences of genomic stress (Australian Pancreatic Cancer Genome Initiative et al., 2013; Snyder et al., 2014; McGranahan et al., 2016; Chan et al., 2019). However, DDR-centered models generally do not integrate repair-pathway state with the epigenetic regulation of antigen presentation and the downstream immune context within a common quantitative framework (Singh et al., 2026). Consequently, DDR provides a mechanistically important genomic dimension of immunogenicity but does not, by itself, capture whether genomic alterations become transcriptionally accessible, presented, and immunologically engaged.

### Conceptual fragmentation and its consequences

Collectively, these approaches capture complementary but incomplete dimensions of tumor immunogenicity: immune-centric models characterize the downstream immune state, epigenetic models define regulatory accessibility, and DDR models describe genomic instability and repair-associated mutational processes. The unresolved problem is therefore not the absence of informative biomarkers or mechanisms within any individual domain, but the lack of a common framework for evaluating how these dimensions interact to shape tumor immune visibility and therapeutic response. This fragmentation may contribute to the context-dependent performance of single biomarkers such as PD-L1 expression and tumor mutational burden and motivates an integrative framework in which epigenetic, DDR, and immune information can be evaluated jointly (Herbst et al., 2014; Topalian et al., 2016; Sharma et al., 2017).

## The central hypothesis: immunogenicity control axis (ICA)

We propose the Immunogenicity Control Axis (ICA) as a directionally organized biological framework integrating epigenetic state, DNA repair pathway configuration, and immune activity. Within this framework, tumor immunogenicity is not an intrinsic or static property but a dynamically regulated output determined by upstream molecular constraints. The ICA therefore specifies a hypothesized directional architecture in which epigenetic regulation and DDR-associated genomic processes influence complementary determinants of tumor immune visibility, which in turn shapes the observable immune state.

This hypothesis is grounded in converging evidence demonstrating that chromatin accessibility precedes transcriptional activation of immune-relevant genes, and that DNA damage response (DDR)-driven mutational processes precede neoantigen formation, thereby providing a mechanistic rationale for testing directional relationships between tumor-intrinsic regulation and immune recognition (Roulois et al., 2015; Ceccaldi et al., 2016; McGranahan et al., 2016; Topper et al., 2017; Singh et al., 2026). By integrating these processes, the ICA framework proposes a testable directional model in which epigenetic and genomic determinants jointly influence tumor immune visibility and the resulting immune state.

The biological hypothesis and its computational operationalization are summarized separately in Figures 1A,B, respectively. The framework integrates three molecular axes: an Epigenetic Axis representing chromatin-mediated transcriptional permissiveness and antigen-presentation capacity; a DDR Pathway Axis representing repair pathway activity and associated mutational processes; and an Immune Axis representing cytolytic activity, checkpoint engagement, and interferon-responsive signaling. Each axis is quantified through pathway- or feature-level measurements and can subsequently be integrated into a composite ICA Score. In its general form, the computational representation is defined as ICA Score = w_1_E + w_2_D + w_3_I, where *E*, *D*, and *I* denote standardized Epigenetic, DDR, and Immune component scores, respectively, and the non-negative weights sum to 1. The primary implementation uses equal weighting as a transparent, outcome-independent reference model rather than as an assumption that the three biological systems contribute equally or interact additively. Accordingly, the ICA Score should be distinguished from the broader biological ICA framework: the score provides a first-order quantitative representation for empirical evaluation, whereas the biological framework permits reciprocal, conditional, and nonlinear interactions among epigenetic regulation, DDR, and immune activity. Natural-compound perturbation is incorporated as a downstream exploratory prioritization layer in which CMap/LINCS-derived signatures are used to identify candidate compounds predicted to oppose ICA-associated transcriptional states (Figure 1).

**FIGURE 1:**
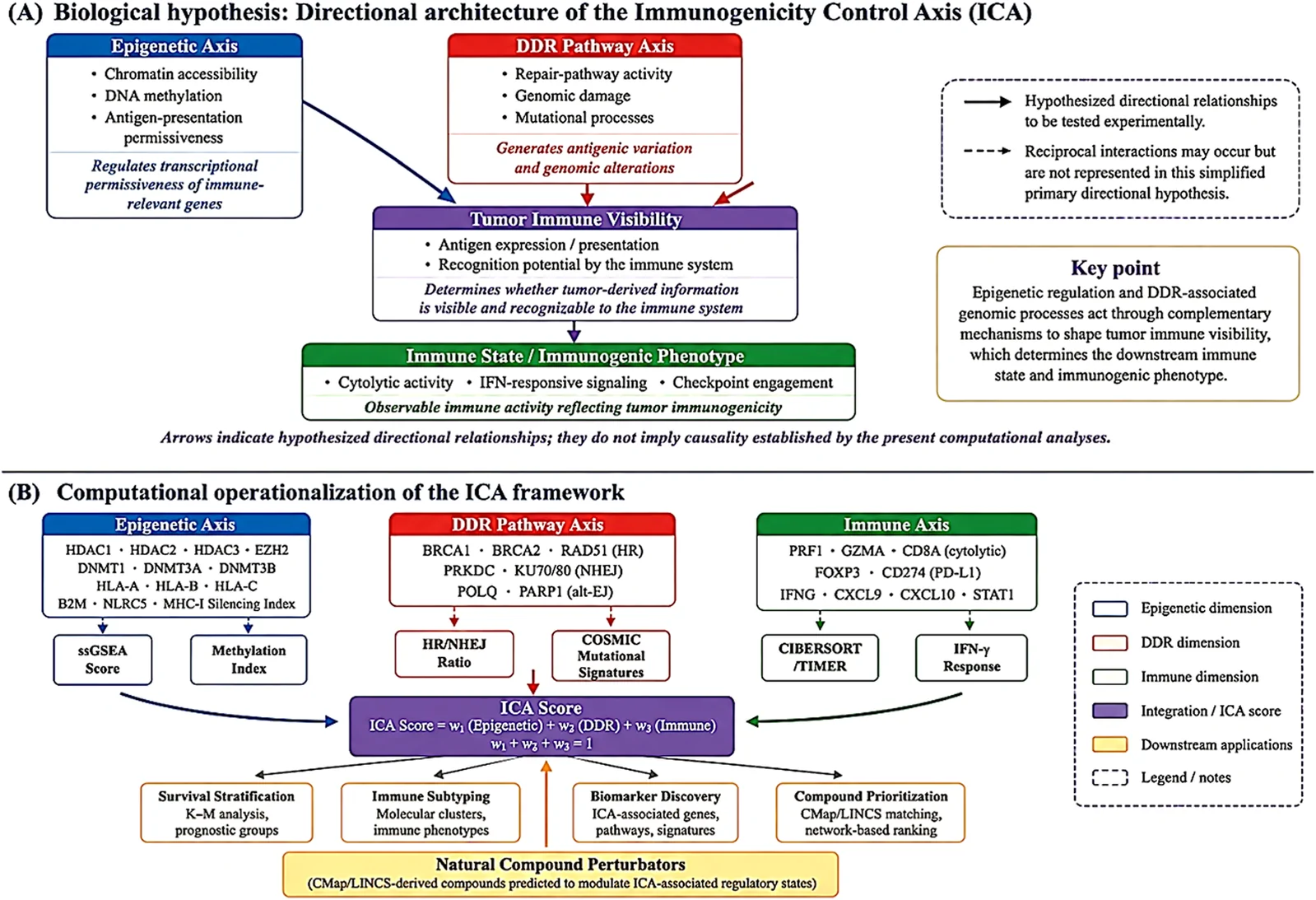
Biological hypothesis and computational operationalization of the Immunogenicity Control Axis (ICA). **(A)** Biological hypothesis. The ICA proposes a directional model in which epigenetic state regulates transcriptional permissiveness and antigen-presentation capacity, while DNA damage response (DDR) state influences repair-associated genomic and mutational processes. These complementary tumor-intrinsic dimensions are hypothesized to converge on tumor immune visibility and thereby influence downstream immune activity, including cytolytic responses, interferon-associated signaling, and checkpoint engagement. Arrows indicate hypothesized directional relationships to be tested experimentally and should not be interpreted as causal relationships established by the present computational analyses. **(B)** Computational operationalization. Epigenetic, DDR, and Immune dimensions are quantified independently using modality-appropriate molecular features and standardized before integration into the ICA Score. In its general form, ICA Score = w_1_E + w_2_D + w_3_I, where *E*, *D*, and *I* denote standardized Epigenetic, DDR, and Immune component scores and the non-negative weights sum to 1. The equal-weight implementation provides the primary outcome-independent reference model. ICA-derived representations can subsequently be tested for associations with molecular stratification and clinical outcomes and compared with established biomarkers, while perturbational signatures can be used for exploratory compound prioritization.

Figure 1A therefore represents the primary directional hypothesis of ICA, whereas Figure 1B defines its computational operationalization; the former specifies biological relationships requiring empirical testing, while the latter provides a quantitative strategy for evaluating those relationships in molecular datasets.

### Epigenetic control layer

The epigenetic layer establishes the transcriptional permissiveness of tumor cells through regulation of chromatin accessibility. Histone deacetylases (HDACs) and DNA methyltransferases (DNMTs) modulate the accessibility of promoters and enhancers associated with immune-relevant loci, including antigen presentation machinery (e.g., MHC class I/II) and interferon-responsive pathways (Seto and Yoshida, 2014; Roulois et al., 2015; Topper et al., 2017; Jones et al., 2019). In addition, epigenetic regulation of DNA repair genes, including O6-methylguanine-DNA methyltransferase (MGMT), further illustrates the close functional interplay between chromatin regulation and DNA repair pathway activity, reinforcing the biological rationale underlying the ICA framework (Gupta et al., 2023; Singh et al., 2025).

A repressive chromatin state restricts transcription of these genes, effectively limiting tumor visibility to immune surveillance. In contrast, epigenetic activation increases chromatin accessibility, enabling transcriptional engagement of immune signaling pathways and enhancing the capacity for antigen presentation (Roulois et al., 2015; Topper et al., 2017). This establishes epigenetic regulation as the upstream boundary condition within the ICA, determining whether downstream immunogenic processes can be engaged.

Within the ICA hypothesis, epigenetic accessibility is therefore considered a permissive constraint on immune-relevant transcription. Silencing of antigen-presentation or interferon-responsive programs may restrict effective immune recognition even when potentially immunogenic genomic alterations are present. Epigenetic state is consequently proposed to influence the capacity for immune responsiveness rather than determine its downstream execution independently of genomic and microenvironmental context.

### DDR pathway configuration and mutational processes

The DDR Axis represents variation in DNA repair pathway activity and associated genomic processes that may influence tumor immunogenicity. DNA double-strand breaks can be resolved through homologous recombination (HR), classical non-homologous end joining (c-NHEJ), and alternative end joining (alt-EJ), which differ in repair template requirements, fidelity, and genomic consequences (Ceccaldi et al., 2016; Jeggo et al., 2016; Kushwah et al., 2024a).

We hypothesize that differences in repair pathway configuration may contribute to variation in the genomic substrate available for immune recognition. Altered HR activity and increased reliance on error-prone end-joining mechanisms can promote distinct forms of genomic alteration; however, the generation of immunogenic neoantigens cannot be inferred directly from repair pathway expression alone. Mutation type, clonality, transcription, peptide processing, HLA binding, and antigen presentation collectively determine whether a genomic alteration becomes immunologically relevant (Australian Pancreatic Cancer Genome Initiative et al., 2013; McGranahan et al., 2016).

Accordingly, the ICA framework does not assign individual COSMIC mutational signatures directly to NHEJ or alt-EJ activity. Instead, independently estimated DDR pathway scores are proposed to be evaluated against mutational signature exposures, mutation burden, and neoantigen-associated features as complementary genomic measurements. A genomic extension of the DDR Axis is therefore the empirical assessment of whether particular repair configurations are associated with reproducible mutational processes.

To examine the genomic component of the DDR Axis, mutational-signature exposures were evaluated in relation to the independently derived DDR scores in the TCGA-CESC analysis. Figure 2 displays the relative contributions of selected single-base substitution (SBS) signatures across evaluable tumors ordered by DDR score. This analysis provides an exploratory patient-derived assessment of the relationship between expression-defined DDR state and mutational processes; it does not establish that a specific DNA-repair pathway directly generates any individual mutational signature or associated immunogenic phenotype.

**FIGURE 2:**
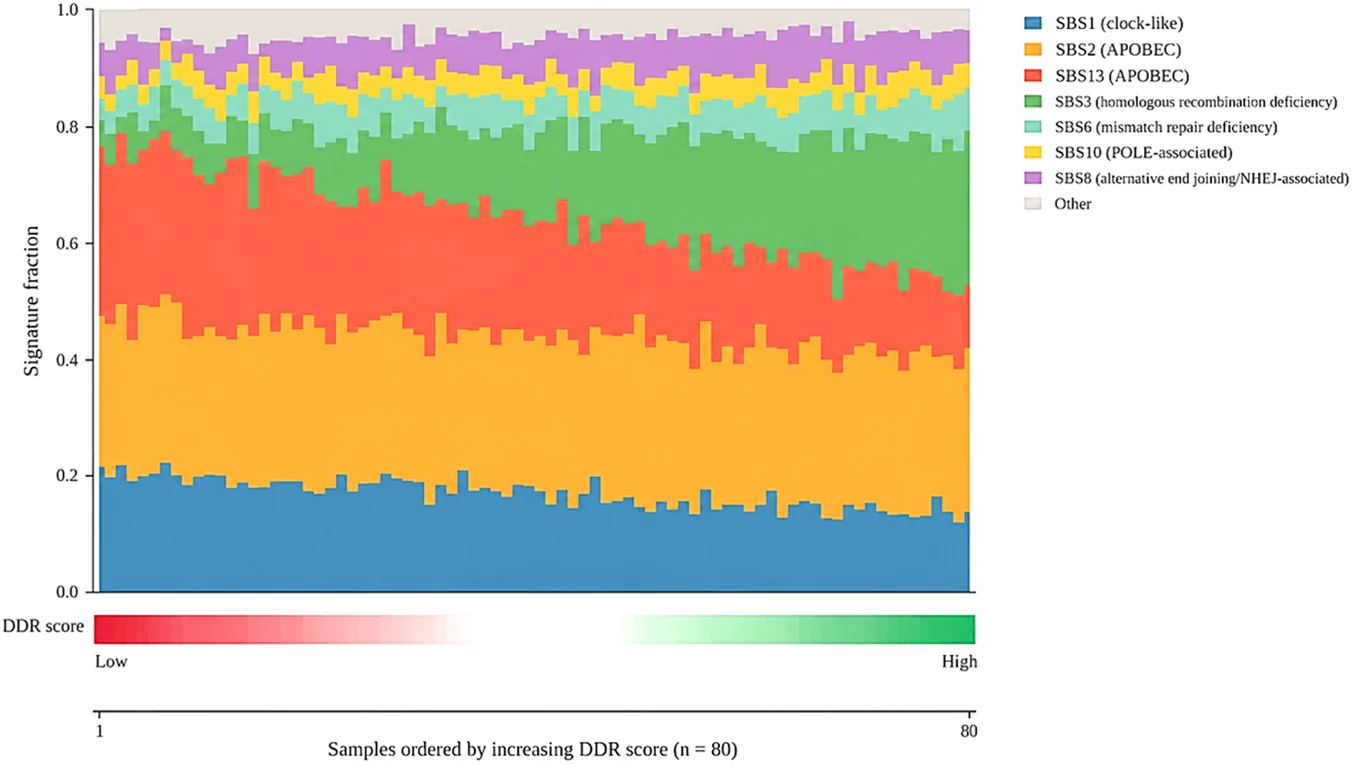
Mutational-signature composition across TCGA-CESC tumors ordered by DNA damage response score. Relative contributions of selected single-base substitution (SBS) signatures are shown across 80 evaluable TCGA-CESC tumors ordered from lower to higher DNA damage response (DDR) scores. Each vertical segment represents one tumor, and the stacked signature fractions sum to 1. The annotation strip below the plot shows the corresponding DDR-score gradient. The displayed signatures include SBS1, SBS2, SBS13, SBS3, SBS6, SBS10, SBS8, and a combined “Other” category. Mutational-signature exposures and DDR scores were derived independently within the computational workflow and are displayed together to examine their distribution across the DDR-score continuum. The visualization is exploratory and does not assign individual SBS signatures directly to specific DNA-repair pathways.

The signatures displayed in Figure 2 represent mutational processes relevant to interpretation of the DDR component of ICA. SBS3 is associated with homologous-recombination deficiency, whereas SBS2 and SBS13 represent APOBEC-associated mutagenesis, a prominent mutational process in cervical cancer. SBS8 is retained as a signature of unresolved aetiology and is not assigned to NHEJ or alt-EJ within the ICA framework. Accordingly, the exploratory analysis evaluates whether independently estimated repair-pathway states show patterns of association with mutational-signature exposures, mutation burden, or neoantigen-associated genomic features; these relationships should not be interpreted as direct evidence that a particular repair pathway generates a specific signature or immunogenic consequence. SBS3 provides a reference signature associated with homologous-recombination deficiency, whereas SBS2 and SBS13 represent APOBEC-associated mutagenesis, a prominent mutational process in cervical cancer. SBS8 is retained as a signature of unresolved aetiology and is not assigned to NHEJ or alt-EJ within the ICA framework. The empirical test is therefore not whether the pattern shown in Figure 2 is reproduced, but whether independently estimated repair-pathway states in patient tumors demonstrate reproducible associations with mutational-signature exposures, mutation burden, or neoantigen-associated genomic features after appropriate adjustment for relevant molecular and clinical covariates.

### Immune activation layer

The immune layer represents the downstream manifestation of ICA regulation and reflects the functional output of upstream epigenetic and DDR processes. Key components include neoantigen presentation, PD-L1 expression, interferon signaling, and T-cell infiltration, all of which are influenced by both transcriptional accessibility and mutational landscape (Snyder et al., 2014; Rooney et al., 2015; Van Allen et al., 2015; McGranahan et al., 2016; Cristescu et al., 2018; Thorsson et al., 2018).

Within this framework, immune activation is not an independent driver but an emergent property determined by the coordinated interaction of upstream regulatory layers. For example, neoantigen presence alone is insufficient to elicit an immune response if antigen presentation pathways are epigenetically silenced. Conversely, transcriptional accessibility without sufficient antigenic diversity may fail to generate effective immune recognition.

This integrated perspective redefines immune responsiveness as a regulated output of tumor-intrinsic processes, rather than a solely immune-mediated phenomenon. By embedding immune activation within the ICA, the model establishes a mechanistic continuum linking chromatin regulation, genomic instability, and immune recognition.

We propose that tumor immunogenicity follows a “Regulatory Convergence Principle,” wherein epigenetic accessibility, DNA repair pathway bias, and immune signaling must align to generate effective immune visibility. Disruption at any layer constrains immunogenic output, while coordinated modulation enables therapeutic reprogramming. Accordingly, molecular stratification, outcome benchmarking, and perturbational compound prioritization are treated as downstream tests or applications of the ICA hypothesis and are not themselves components of its biological definition.

### Natural compounds as multi-axis ICA perturbators

Within the proposed Immunogenicity Control Axis (ICA), natural compounds are conceptualized as candidate multi-axis perturbators rather than empirically selected adjunctive agents. This distinction is central to the framework. The biological relevance of a compound is not inferred from activity against an isolated molecular target but from its potential to influence regulatory processes distributed across the epigenetic, DNA damage response (DDR), and immune axes. The ICA hypothesis therefore provides a systems-level rationale for evaluating natural compounds according to the breadth and direction of their molecular perturbation (Shankar et al., 2016; Farhan et al., 2019; Hassan et al., 2019).

At the epigenetic level, several naturally derived compounds have been reported to influence histone acetylation, DNA methylation, and chromatin-associated transcriptional programs (Shankar et al., 2016; Hassan et al., 2019). Curcumin and resveratrol, for example, have been associated with modulation of DNMT-, HDAC-, and other chromatin-regulatory processes in experimental cancer models (Shankar et al., 2016; Farhan et al., 2019; Hassan et al., 2019). Such effects are relevant to the ICA because chromatin state can determine the transcriptional accessibility of antigen-presentation and interferon-responsive programs (Roulois et al., 2015; Topper et al., 2017; Jones et al., 2019). However, epigenetic modulation alone is unlikely to define tumor immune visibility; its functional consequence depends on the genomic and immune context in which the perturbation occurs.

Natural compounds may also alter replication stress, DNA damage signaling, and repair-associated processes (Lagunas-Rangel and Bermúdez-Cruz, 2020). Within the ICA model, these effects are interpreted in relation to DDR pathway configuration rather than generalized genomic instability. Perturbation of homologous recombination, classical non-homologous end joining, or alternative end joining could alter the balance among repair fidelity, genomic diversification, and damage-associated immune signaling (Ceccaldi et al., 2016; Jeggo et al., 2016; Pilié et al., 2019). The direction and immunological consequence of such perturbation are expected to be tumor- and compound-context dependent and therefore require pathway-level assessment rather than assumptions based on compound identity alone.

A third level of potential ICA perturbation involves inflammatory, interferon-responsive, and immune-checkpoint-associated signaling. Natural products and phytochemicals have been reported to influence cytokine/chemokine networks and immune-checkpoint-associated pathways in experimental cancer systems (Gupta et al., 2022; Huo et al., 2022). Within the ICA framework, these immune-associated effects are considered jointly with tumor-intrinsic epigenetic and DDR states. A compound that alters an immune-associated transcriptional program without restoring antigen-presentation capacity or modifying an adverse repair-associated state may therefore produce only partial ICA perturbation.

The central proposition is that natural compounds can be evaluated as candidate system-level perturbators of ICA-associated molecular states. This does not imply that transcriptional or network connectivity establishes direct target engagement or therapeutic efficacy. Instead, the ICA framework generates a testable prioritization strategy: compounds predicted to induce coordinated and directionally favorable changes across more than one ICA axis should be prioritized for mechanistic validation over compounds producing isolated single-axis effects. Within the proposed ICA framework, this prioritization principle can be computationally operationalized through perturbation-signature matching and network-based analysis.

## Computational methods and ICA implementation

### Data acquisition and preprocessing

The following methods operationalize the ICA hypothesis for reproducible computational evaluation and are presented separately from its biological formulation to preserve the distinction between framework definition and analytical implementation. Publicly available molecular and clinical data from The Cancer Genome Atlas Cervical Squamous Cell Carcinoma and Endocervical Adenocarcinoma project (TCGA-CESC) were acquired through the NCI Genomic Data Commons (GDC) using the TCGAbiolinks R package. Primary-tumor data comprised STAR-processed RNA-sequencing data, Illumina HumanMethylation450 DNA methylation beta values, masked somatic mutation calls, and available clinicopathological annotations. RNA-sequencing counts were retained for differential-expression analyses, whereas FPKM values were log2-transformed [log2(FPKM +1)] for single-sample gene-set scoring. Ensembl identifiers were mapped to gene symbols using the corresponding Summarized Experiment annotation, and duplicated gene symbols were collapsed by their mean expression. DNA methylation and somatic mutation data were processed independently before patient-level integration using TCGA identifiers. Overall-survival information and available clinicopathological variables were obtained from the corresponding TCGA-CESC clinical records.

### Construction of the epigenetic axis

Epigenetic-state features were quantified by single-sample gene-set enrichment analysis (ssGSEA) of prespecified transcriptional programs representing histone deacetylase/DNA methyltransferase activity, MHC class I and II antigen-presentation machinery, type I interferon and IFN-γ-associated responses, and chromatin-opening and chromatin-silencing programs (Barbie et al., 2009; Hänzelmann et al., 2013). Gene-set scores were calculated from log2(FPKM +1)-transformed expression data and standardized before integration. The expression-derived Epigenetic Axis was oriented such that higher values represented a relatively permissive, immune-associated transcriptional state: standardized MHC-I, MHC-II, IFN-γ, type I interferon, and open-chromatin scores contributed positively, whereas HDAC, DNMT, and closed-chromatin scores contributed negatively. The complete gene composition, source, author adaptations, scoring direction, and analytical role of each prespecified Epigenetic signature are provided in Supplementary Table S1 and in the corresponding analysis script.

DNA methylation was evaluated separately at promoter-associated CpG probes mapped to selected antigen-presentation, interferon-signalling, immune-regulatory, and epigenetic genes using Illumina HumanMethylation450 annotations. Promoter probes were defined by TSS200/TSS1500 annotation, and gene-level methylation summaries were derived from available beta values. The methylation component was used as a complementary measure of epigenetic repression rather than as a direct surrogate for chromatin accessibility.

#### Construction of the DNA damage response axis

DDR pathway states were quantified by ssGSEA using prespecified gene sets representing homologous recombination (HR), classical non-homologous end joining (c-NHEJ), alternative end joining (alt-EJ), mismatch repair (MMR), nucleotide-excision repair, base-excision repair, Fanconi-anemia-associated repair, and DNA-damage checkpoint signalling. For the primary DDR Axis, HR, c-NHEJ, and alt-EJ scores were standardized and integrated as

$${DDR}_{i}=\frac{z\left(c{NHEJ}_{i}\right)+z\left(alt{EJ}_{i}\right)}{2}-z\left({HR}_{i}\right),$$

such that higher values represented a relative transcriptional shift toward end-joining-associated versus HR-associated repair programs. Importantly, this score represents relative pathway-associated transcriptional state and does not directly measure DNA-repair fidelity, pathway flux, or functional repair capacity. The complete gene composition, source, author adaptations, scoring direction, and analytical role of each prespecified DDR signature are provided in Supplementary Table S1 and in the corresponding analysis script.

Somatic mutational patterns were analyzed separately as genomic correlates of DDR state. Trinucleotide mutation matrices were generated from TCGA-CESC somatic mutation calls and decomposed into mutational signatures, with particular attention to signatures associated with homologous-recombination deficiency, APOBEC activity, and mismatch-repair deficiency. Relationships between DDR-axis scores and mutational-signature features were treated as associations rather than evidence that expression-defined repair states caused individual mutations or neoantigens.

### Construction of the immune axis

Immune state was characterized using complementary transcriptional and deconvolution-based features. ssGSEA was applied to prespecified signatures representing cytolytic activity, T-cell-inflamed/IFN-γ-associated responses, CD8^+^ T-cell activity, T-cell exhaustion, regulatory T cells, natural killer cells, macrophage-associated states, stromal exclusion, immune checkpoints, and tertiary-lymphoid/B-cell-associated programs. Expression of individual immune markers, including CD274 (PD-L1), PDCD1, CTLA4, LAG3, HAVCR2, TIGIT, CD8A, FOXP3, PRF1, GZMA, GZMB, and IFNG, was retained for complementary analyses. The complete gene composition, source, author adaptations, scoring direction, and analytical role of each prespecified Immune signature are provided in Supplementary Table S1 and in the corresponding analysis script.

Immune-cell composition was additionally estimated from bulk transcriptomic data using deconvolution approaches implemented through immunedeconv, including EPIC, xCell, and MCP-counter where computationally available. The resulting features were interpreted as inferred immune-cell enrichment or abundance estimates rather than direct measurements of tumor-infiltrating leukocytes. The composite Immune Axis was oriented such that increasing values represented a relatively immune-active tumor state.

#### ICA Score construction and alternative integration

Epigenetic, DDR, and Immune axis scores were harmonized by TCGA patient identifier and standardized before integration. The prespecified primary ICA Score for patient was defined as the arithmetic mean of the three standardized component scores:

$$\text{IC}A_{i}=\left(E_{i}+D_{i}+I_{i}\right)/3$$

Where, and denote the standardized Epigenetic, DDR, and Immune axis scores, respectively. Equal weighting was selected as a transparent, parsimonious, and outcome-independent reference implementation. It was not intended to imply that the three biological systems contribute equally to tumor immunogenicity or that their underlying relationships are biologically additive. Equal weighting was therefore treated as a prespecified null integration model against which alternative data-driven representations could be compared, rather than as a biologically optimized formulation of tumor immunogenicity.

To examine the dependence of ICA representation on the integration strategy, two alternative approaches were implemented as exploratory sensitivity analyses: an unsupervised principal-component-based score derived from the first principal component of the three standardized axes and an outcome-supervised elastic-net Cox model. The equal-weight score remained the primary ICA implementation because its definition is independent of clinical outcome. The ICA Score should therefore be distinguished from the broader ICA biological framework: the former is a first-order quantitative summary, whereas the latter permits reciprocal, conditional, and nonlinear interactions among epigenetic regulation, DDR, and immune activity.

#### Unsupervised molecular-state analysis

To investigate whether the three ICA dimensions organize tumors into reproducible multi-axis states, standardized ICA component features were subjected to unsupervised analysis. Consensus clustering was evaluated using 1,000 resampling iterations, 80% item resampling, Pearson-distance hierarchical clustering, and a fixed random seed. Cluster stability was assessed using the proportion of ambiguous clustering (PAC), with lower PAC values indicating greater stability. Non-negative matrix factorization and dimensionality-reduction approaches were used as complementary exploratory procedures for class discovery and visualization. Molecular states identified through these analyses were regarded as exploratory computational strata and not as clinically validated cervical-cancer subtypes.

### Generation of the synthetic ICA feature matrix

A synthetic matrix comprising 30 ICA-associated genes and 80 profiles was generated solely to illustrate predefined multi-axis molecular patterns. Ten genes were assigned to each of the Immune, DDR, and Epigenetic modules. Profiles were allocated in advance to immune-dominant (n = 20), epigenetically repressed (n = 25), DDR-associated (n = 20), and low-activity (n = 15) states. Using a fixed random seed of 42, values were sampled independently from Gaussian distributions with a residual standard deviation of 0.70. State-specific means were assigned as follows: the immune-dominant state, Immune = 1.50, DDR = −0.25, epigenetic repressors = −0.50, and antigen-presentation genes = 0.75; the epigenetically repressed state, Immune = −0.75, DDR = 0, epigenetic repressors = 1.50, and antigen-presentation genes = −1.50; the DDR-associated state, Immune = 0.10, DDR = 1.50, epigenetic repressors = 0, and antigen-presentation genes = −0.15; and the low-activity state, Immune = −0.75, DDR = −0.65, epigenetic repressors = −0.35, and antigen-presentation genes = −0.75. Values were subsequently standardized independently for each gene and limited to the displayed range of −3 to +3. HPV status and FIGO stage were generated independently of the expression matrix using categorical sampling probabilities of 0.80/0.20 for HPV-positive/HPV-negative status and 0.35/0.25/0.25/0.15 for FIGO stages IB/IIA/IIB/IIIB, respectively; these annotations were included solely to demonstrate annotation tracks and were not used to generate the molecular states. The complete generating script, parameter table, synthetic matrix, and annotation file are provided in the accompanying GitHub repository.

#### Survival and prognostic benchmarking

Overall-survival analyses were restricted to patients with available survival-time and event information. The ICA Score was evaluated as a continuous standardized variable and, for Kaplan–Meier visualization, using a median-based categorical representation. The 304 evaluable TCGA-CESC cases were dichotomized using the cohort median ICA Score: values greater than or equal to the median were classified as ICA-high (n = 152), whereas values below the median were classified as ICA-low (n = 152). Kaplan–Meier estimates and log-rank tests were used to compare survival distributions, and Cox proportional-hazards regression was used to evaluate associations between overall survival and the ICA Score, its individual component axes, and comparator biomarkers. Tumor mutational burden (TMB) and CD274/PD-L1 expression were evaluated as molecular comparators. Multivariable Cox models incorporated available clinicopathological covariates, including age and disease stage where data completeness permitted, and concordance-based measures were used for comparative prognostic assessment.

For the exploratory 3-year overall-survival classification analysis, survival status at 36 months was used as the prespecified binary endpoint. Patients with an observed death at or before 36 months were classified as events, whereas patients known to be alive beyond 36 months were classified as non-events; patients censored before 36 months without a documented event were excluded from the binary ROC analysis. Five feature–model combinations were compared: an ICA composite model using XGBoost; an ICA-components model incorporating the Epigenetic, DDR, and Immune Axis scores using elastic-net logistic regression; a combined TMB + PD-L1 model using XGBoost; TMB alone using elastic-net logistic regression; and PD-L1 expression alone using elastic-net logistic regression. Missing predictor values were imputed using the corresponding median, and predictors were standardized before model fitting. Discrimination was evaluated using stratified five-fold cross-validation with a fixed random seed of 42. The mean cross-validated area under the receiver operating characteristic curve was used for model comparison. These estimates represent internally cross-validated performance within TCGA-CESC and not independent external validation.

These analyses were considered exploratory evaluations of prognostic association and computational feasibility rather than clinical validation of the ICA Score. TCGA-CESC survival associations were not interpreted as evidence of immunotherapy-response prediction because treatment-specific predictive utility requires independent cohorts with documented treatment exposure and response endpoints.

### Mutational-signature and neoantigen-related analysis

Somatic mutation data were used to characterize genomic features potentially linking DDR configuration with immunogenic substrate. Trinucleotide mutation contexts and mutational-signature contributions were derived computationally and compared with established COSMIC single-base-substitution signatures, with emphasis on patterns associated with homologous-recombination deficiency, APOBEC activity, and mismatch-repair deficiency. Associations between expression-defined DDR states, mutational signatures, mutation burden, and neoantigen-related features were evaluated as hypothesis-generating relationships. Neither mutational-signature exposure nor repair-pathway expression was considered sufficient by itself to establish the generation of immunogenic neoepitopes.

### Perturbational signature analysis and compound prioritization

To generate an ICA-associated perturbational query, differential-expression analysis was performed between ICA-high and ICA-low TCGA-CESC tumors using edgeR TMM normalization followed by the voom–limma framework. Genes with a false-discovery-rate-adjusted P value <0.05 were ranked by the moderated t-statistic, and up to 150 positively and 150 negatively associated genes were retained as the bidirectional query. The reported analysis used the LINCS L1000 GSE92742 Level 5 COMPZ.MODZ dataset accessed locally through cmapR; the alternative CLUE implementation was not used for Figure 3. Query genes were restricted to available L1000 landmark genes. For each perturbational signature, landmark-gene values were converted to normalized ranks; the mean centered rank was calculated separately for the upregulated and downregulated query sets, and the directional connectivity score was defined as one-half of their difference. Negative scores therefore indicate transcriptional opposition to the ICA-associated query. Where a compound had multiple available profiles, the median signature-level score was used for compound-level ranking. Cell line, dose, and exposure duration were retained as contextual metadata but were not assigned causal or context-independent interpretations. The analysis is consequently considered hypothesis-generating and not evidence of restoration of tumor immunogenicity, target engagement, or therapeutic activity.

**FIGURE 3:**
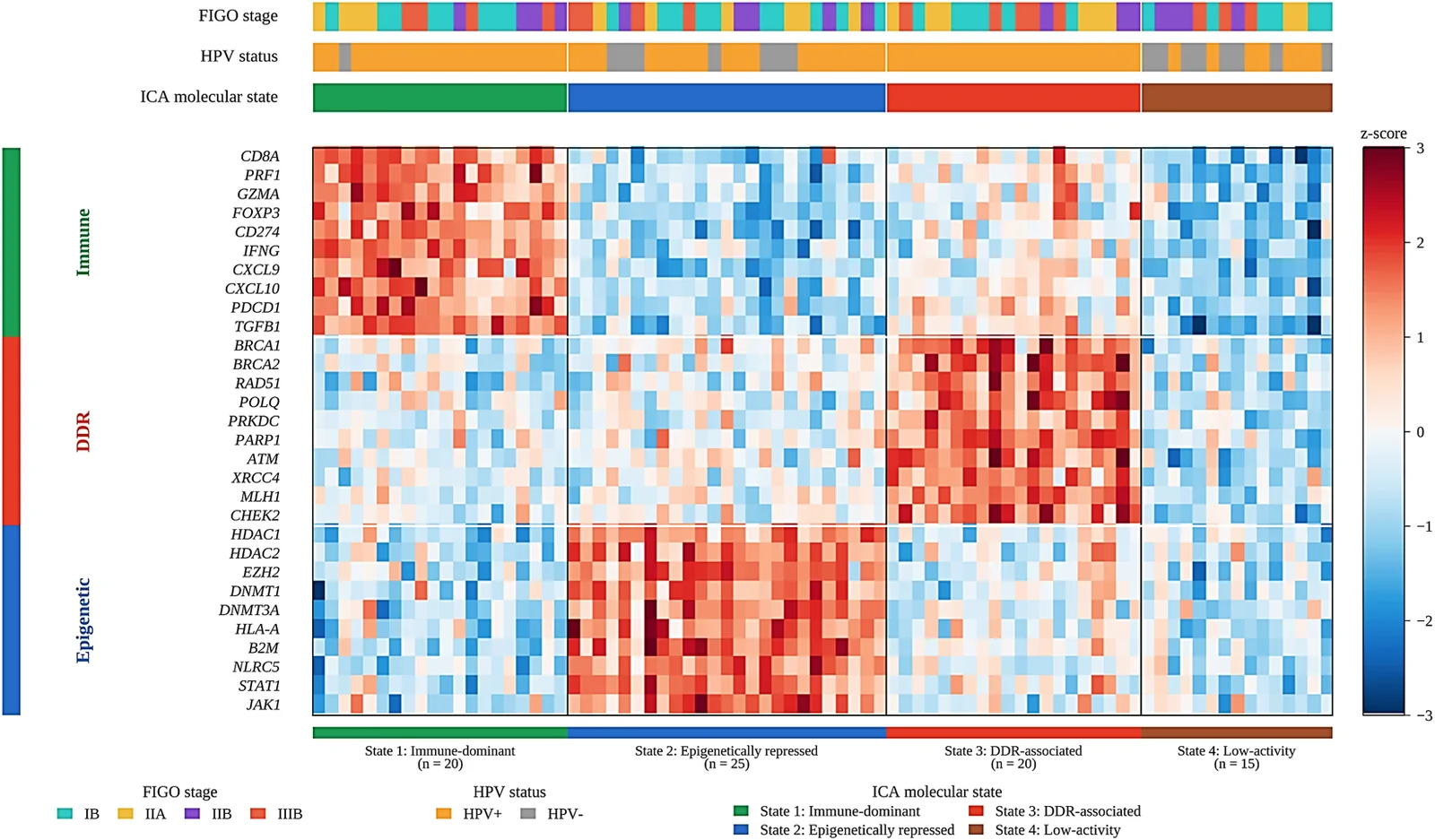
Synthetic demonstration of predefined Immunogenicity Control Axis (ICA) molecular states. Heatmap showing gene-wise standardized expression (z-scores) of a 30-gene ICA feature set across 80 simulated profiles. Four patterns were predefined: State 1 (Immune-dominant, n = 20), State 2 (Epigenetically repressed, n = 25), State 3 (DDR-associated, n = 20), and State 4 (Low-activity, n = 15). Values were generated from Gaussian distributions using the state- and module-specific means and residual standard deviation described in the Methods, with a fixed random seed of 42, followed by independent gene-wise standardization and display over −3 to +3. FIGO-stage and HPV-status tracks were simulated independently using prespecified categorical probabilities and were not used to generate the molecular patterns. The complete script, parameter table, matrix, and annotation file are provided in the accompanying GitHub repository.

Natural-compound candidates were defined by intersection with a prespecified curated list of plant-derived or naturally occurring compounds with reported relevance to at least one ICA dimension. Candidates with negative compound-level connectivity scores were retained and ordered from the most negative to the least negative score. The ranking table was subsequently annotated with the number of ICA-associated network nodes linked to each compound and the Epigenetic, DDR, and Immune dimensions represented by those associations. No P-value threshold was applied to the connectivity ranking. The complete query signature, compound-level ranking table, and Figure 3 plotting data are provided in the accompanying GitHub repository.

### Network-based prioritization

Candidate compounds, compound–target associations obtained from the implemented network-analysis workflow, and ICA-associated genes were integrated with protein–protein interaction information to construct a multilayer compound–target–ICA network. Network topology was used to identify candidate perturbators and molecular nodes spanning more than one ICA regulatory dimension. Network centrality or cross-axis connectivity was interpreted as a prioritization feature rather than evidence that a compound simultaneously modulates all connected targets in a biological system. Experimental target-engagement and perturbation studies remain necessary to establish multi-axis activity.

#### Computational reproducibility

The complete analysis pipeline is publicly available at the accompanying GitHub repository [https://github.com/Dr-SS03/ica-score-cervical-cancer-pipeline]. Scripts are organized sequentially for TCGA-CESC data acquisition, Epigenetic-, DDR-, and Immune-axis construction, ICA integration and clustering, survival analysis, mutational-signature and neoantigen-related analysis, CMap/LINCS perturbational querying, network analysis, and exploratory predictive modeling. Public source datasets are retrieved from their original repositories rather than redistributed. Intermediate and derived analytical objects are generated locally during pipeline execution. This separation of source data, analytical code, and derived outputs was adopted to facilitate independent inspection and reproduction of the computational workflow. The repository additionally contains Figure 4 generating script and parameter files, Supplementary Table S1 source definitions, and Figure 3 query signature, compound-level ranking table, and plotting data.

**FIGURE 4:**
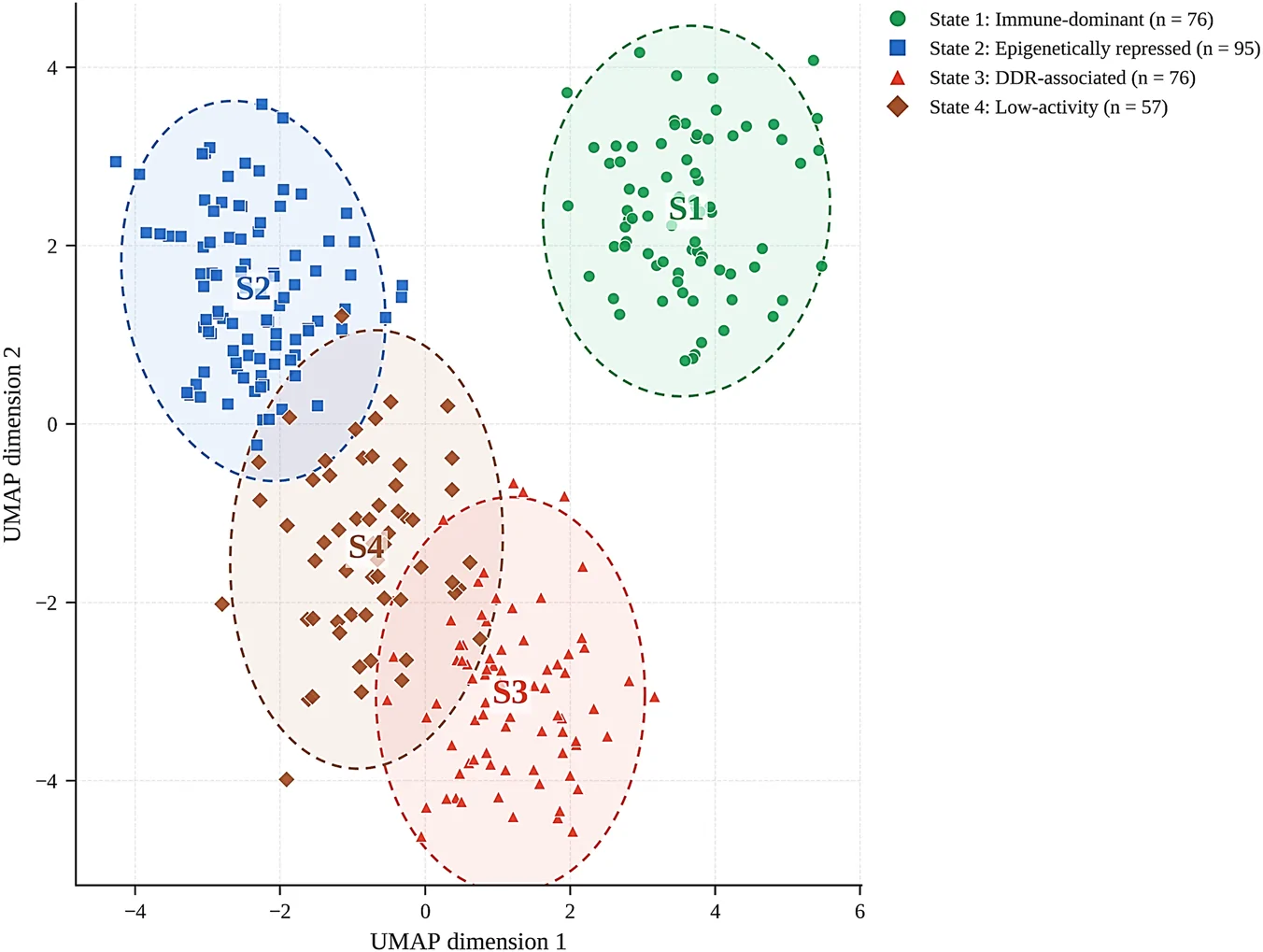
Exploratory UMAP representation of ICA-associated molecular states in TCGA-CESC. Uniform Manifold Approximation and Projection (UMAP) of the 30-gene Immunogenicity Control Axis (ICA) feature matrix from 304 evaluable TCGA-CESC tumors. Each point represents an individual tumor and is annotated according to the molecular-state assignment obtained from the unsupervised ICA analysis: State 1 (Immune-dominant, n = 76), State 2 (Epigenetically repressed, n = 95), State 3 (DDR-associated, n = 76), and State 4 (Low-activity, n = 57). Ellipses represent 95% confidence regions and are included solely to visualize within-state dispersion; they do not define cluster membership or boundaries. The projection provides an exploratory visualization of ICA-associated feature-space structure and should not be interpreted independently as evidence of biological subtype validity, clinical utility, or external reproducibility.

### Genomics-guided operationalization and ICA stratification

The analyses presented below illustrate how the ICA framework can be operationalized computationally and evaluated across complementary levels of evidence. These include exploratory analyses of publicly available TCGA-CESC molecular and clinical data and, where explicitly indicated, synthetic demonstrations used to illustrate specific analytical procedures. Patient-derived analyses are interpreted as exploratory computational proof of principle, rather than independent biological or clinical validation, whereas synthetic analyses are used solely to demonstrate analytical behavior and are not considered evidence supporting the ICA hypothesis. The provenance and evidentiary status of each analysis are identified in the corresponding text and figure legends, with computational procedures detailed in the preceding Computational Methods and ICA Implementation section.

This implementation provides an operational bridge between the biological ICA hypothesis and patient-level molecular characterization. The Epigenetic, DDR, and Immune component scores represent complementary dimensions of tumor molecular state rather than direct measurements of epigenetic enzyme activity, DNA-repair pathway flux, or immune-cell function. Their integration is therefore used to test whether coordinated multi-axis information captures molecular variation beyond that represented by the individual components, while preserving the distinction between a computational representation of the ICA and direct mechanistic validation of its proposed biological relationships.

Within this framework, the three ICA dimensions are first evaluated separately to preserve their biological interpretability before integration. Epigenetic-state features capture transcriptional and methylation-associated regulation of immune-relevant programs; the DDR dimension represents relative repair-pathway-associated transcriptional states together with complementary genomic features; and the Immune dimension captures transcriptional and deconvolution-derived measures of immune activity. Integration occurs only after axis-specific construction and standardization, thereby preventing the composite ICA Score from obscuring the contribution of its constituent biological dimensions.

Large-scale cancer genomics resources provide an appropriate setting in which to evaluate this multi-axis hypothesis because transcriptomic, epigenomic, genomic, and clinical information can be interrogated within the same disease context. TCGA-CESC therefore serves as the primary patient-derived dataset for exploratory operationalization of the ICA framework, while appropriately matched Gene Expression Omnibus (GEO) datasets may support component-level external evaluation where the requisite molecular and clinical variables are available (The Cancer Genome Atlas Research Network et al., 2013; Hoadley et al., 2014; The Cancer Genome Atlas Research Network, 2017; Thorsson et al., 2018). Importantly, differences in assay platform, feature availability, and clinical annotation preclude assuming that every ICA component can be reconstructed identically across external cohorts.

Biologically, the Epigenetic Axis represents transcriptional and methylation-associated states capable of modifying immune-relevant gene accessibility and antigen-presentation competence. It incorporates HDAC- and DNMT-associated transcriptional programs together with methylation and expression features related to HLA class I, B2M, NLRC5, and associated antigen-presentation machinery (Seto and Yoshida, 2014; Jones et al., 2019; Singh et al., 2025). These features are interpreted as molecular correlates of epigenetic permissiveness or repression rather than direct measurements of chromatin accessibility or epigenetic-enzyme activity.

The DDR Axis represents relative repair-pathway-associated states, particularly the balance among homologous recombination (HR), classical non-homologous end joining (c-NHEJ), and alternative end joining (alt-EJ). Expression-derived repair programs are complemented by genomic features, including mutational-signature patterns, where matched somatic mutation data are available (Ceccaldi et al., 2016; Jeggo et al., 2016; Kushwah et al., 2024a). Importantly, these transcriptional and genomic features characterize DDR-associated molecular states but do not directly measure repair-pathway flux or establish that a particular repair mechanism generated an individual mutation or immunogenic neoantigen.

The Immune Axis represents the downstream immune-associated state of the tumor microenvironment through complementary measures of cytolytic activity, interferon-responsive transcription, checkpoint-associated expression, and computationally inferred immune-cell composition (Rooney et al., 2015; Ayers et al., 2017; Cristescu et al., 2018). Because these measures are derived predominantly from bulk molecular profiles, they are interpreted as immune-associated transcriptional or inferred cellular states rather than direct functional measurements of antitumor immunity.

At the individual-tumor level, axis-specific molecular features are transformed into standardized component scores as described in the Computational Methods and ICA Implementation section. A critical requirement for cross-axis integration is directional harmonization: increasing values must have an explicit and consistent biological interpretation before the components are combined. In the present implementation, the Epigenetic and Immune axes are oriented toward relatively permissive and immune-active states, respectively, whereas the DDR Axis represents a relative shift toward end-joining-associated versus HR-associated transcriptional programs. This orientation prevents differences in score direction from producing an uninterpretable composite and preserves the biological meaning of each component after integration (Barbie et al., 2009; Hänzelmann et al., 2013).

Conceptually, the equal-weight ICA Score is intended to provide a deliberately simple reference representation rather than a mechanistic equation for tumor immunogenicity. Epigenetic regulation, DDR, and immune activity can interact reciprocally and conditionally, and their biological effects may therefore be nonlinear even when their standardized computational representations are summarized additively. The value of the primary score consequently lies in providing an interpretable, outcome-independent baseline against which more complex integration strategies can be tested. A nonlinear or interaction-based model would be favored only if it demonstrates reproducible incremental information beyond this simpler reference formulation in independent data (Riley et al., 2020).

To test whether ICA representation depends materially on this initial specification, unsupervised principal-component-based integration and outcome-supervised elastic-net weighting were implemented as exploratory sensitivity analyses (Zou and Hastie, 2005; Simon et al., 2011). These alternatives are not used to redefine the primary ICA Score but provide explicit comparators for evaluating whether data-driven weighting adds reproducible information beyond the prespecified equal-weight reference. More complex interaction or nonlinear formulations would require the same independent evaluation before being incorporated into the framework (Zou and Hastie, 2005; Simon et al., 2011; Suresh et al., 2022).

Any future outcome-informed optimization of ICA weights should be analytically separated from definition of the primary framework. Model coefficients should be estimated exclusively within a training dataset using a prespecified multivariable or penalized-regression procedure, fixed before evaluation, and subsequently assessed in independent validation data. This separation between framework specification, model development, and external evaluation is necessary to reduce overfitting and optimistic estimates of model performance, particularly in high-dimensional biomarker studies (Collins et al., 2015).

Unsupervised analysis was subsequently used to examine whether coordinated variation across ICA dimensions could organize tumors into reproducible molecular states. As detailed in the Computational Methods and ICA Implementation section, consensus clustering was applied across alternative cluster solutions, with complementary class-discovery and dimensionality-reduction approaches used to assess and visualize molecular structure. Importantly, the number and composition of ICA-associated states were not prescribed by the biological hypothesis. Cluster solutions were evaluated using resampling-based stability criteria rather than selected on the basis of clinical separation alone (Wilkerson and Hayes, 2010). Clinical attributes, including FIGO stage, HPV status, treatment information, and outcome where available, were considered downstream annotations for biological interpretation and association testing rather than variables used to define the molecular states. This separation reduces circularity between molecular-state derivation and subsequent assessment of clinical relevance.

Separately from the patient-derived analyses, a reproducibly generated synthetic feature matrix comprising 30 ICA-associated genes across 80 simulated profiles was used solely to illustrate the analytical architecture of multi-axis ICA stratification. Four molecular patterns were specified before data generation: immune-dominant, epigenetically repressed, DDR-associated, and low-activity states. Values were generated using the Gaussian simulation, state-specific effect parameters, residual-noise structure, fixed random seed, and independent HPV-status and FIGO-stage annotation procedures described in the Methods (Figure 4). The four states were predefined rather than discovered by unsupervised analysis. Accordingly, Figure 4 does not represent TCGA-CESC measurements, empirical identification of cervical-cancer subtypes, estimates of subtype prevalence, or evidence that these states occur in patient populations.

As illustrated in Figure 4, coordinated variation across ICA-associated features can generate distinguishable axis-dominant expression architectures. The predefined immune-dominant state displays relatively increased cytolytic and interferon-responsive features, whereas the epigenetically repressed state is represented by increased chromatin-regulatory features accompanied by reduced antigen-presentation-associated expression. The DDR-associated state demonstrates increased expression of selected repair-pathway components, while the low-activity state lacks a dominant axis-level signal. These patterns were intentionally encoded in the synthetic demonstration and should therefore be interpreted as illustrations of contrasting ICA configurations rather than evidence of biological subtype discovery. Whether analogous molecular states emerge reproducibly in cervical cancer requires their empirical derivation and validation in appropriately characterized patient cohorts.

Having illustrated contrasting ICA configurations using predefined synthetic states, we next examined patient-derived TCGA-CESC profiles independently to assess the structure of ICA-associated molecular variation in reduced-dimensional feature space. Uniform Manifold Approximation and Projection (UMAP) was applied to the ICA feature matrix from 304 evaluable TCGA-CESC tumors, with individual tumors annotated according to molecular-state assignments obtained from the unsupervised analytical workflow (Figure 5). The resulting projection provides an exploratory visualization of ICA-associated feature-space structure and is presented as computational proof of principle rather than independent evidence of biological subtype validity, clinical utility, or external reproducibility. Accordingly, the UMAP representation is used only to visualize the sample distribution of the patient-derived ICA profiles; evidence that these assignments constitute reproducible biological or clinically informative molecular states requires quantitative stability assessment, independent cohort replication, and subsequent biological validation.

**FIGURE 5:**
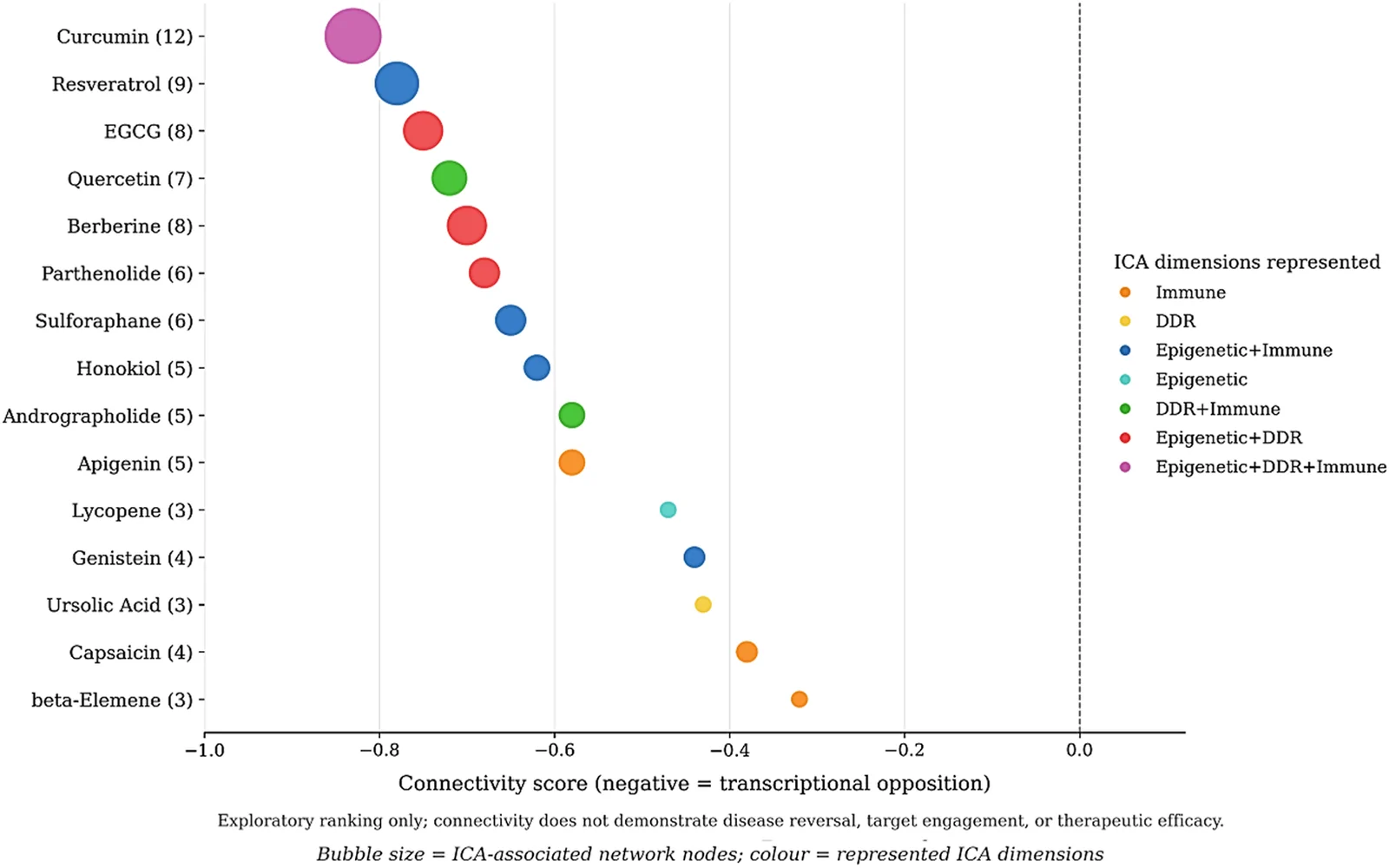
Exploratory prioritization of natural compounds by inverse connectivity to an ICA-associated transcriptional signature. Candidate compounds are ordered according to compound-level connectivity scores generated using the locally implemented LINCS L1000 GSE92742 Level 5 workflow, with more negative values indicating stronger transcriptional opposition to the ICA-associated query. Bubble size represents the number of ICA-associated network nodes linked to each compound, and bubble colour denotes the ICA dimensions represented by those associations. The dashed line at zero separates negative from positive connectivity; no statistical-significance threshold was applied. The complete query signature, ranking table, and plotting data are provided in the accompanying GitHub repository.

The UMAP representation visualizes the distribution of the ICA-associated molecular-state assignments within the reduced-dimensional feature space (Figure 5). Distances and relative positions within this two-dimensional embedding are not interpreted as mechanistic trajectories, biological intermediates, or therapeutic relationships. Moreover, because dimensionality reduction provides visualization rather than independent validation of molecular states, confirmation of ICA-based stratification requires quantitative cluster-stability assessment and replication in independent patient cohorts before biological or clinical labels can be considered established.

The translational premise of ICA stratification is therefore testable rather than assumed. Specifically, the framework predicts that integration of epigenetic, DDR, and immune information will provide incremental prognostic or treatment-response information beyond the individual ICA axes and commonly used biomarkers such as PD-L1 expression and tumor mutational burden (TMB), whose predictive performance is context dependent and incomplete when considered in isolation (Sharma et al., 2017; Cristescu et al., 2018; Thorsson et al., 2018). Evaluation of this hypothesis requires direct benchmarking of the ICA Score against its individual component scores and comparator biomarkers within identical patient cohorts and using identical outcome definitions. For prognostic applications, discrimination and calibration should be evaluated alongside multivariable adjustment for established clinicopathological covariates. For treatment-response applications, evaluation should be restricted to cohorts with documented therapeutic exposure and prespecified response endpoints, with model development and external validation performed separately according to established prediction-model principles (Collins et al., 2015; Efthimiou et al., 2024). Accordingly, the ICA Score is proposed as a candidate integrative molecular stratification metric and testable computational representation of the broader ICA framework, rather than as an established prognostic or predictive biomarker.

### Bioinformatics-driven prioritization of natural compound perturbators

As a downstream test of the perturbational utility of ICA, the framework can be extended from molecular characterization to hypothesis-generating compound prioritization. The implemented workflow prioritizes natural compounds whose perturbational transcriptional profiles oppose the ICA-associated query signature. The objective of this analysis is hypothesis-generating compound prioritization rather than demonstration of pharmacological efficacy, target engagement, or immunomodulatory activity. Although the ICA framework is applicable to broader pharmacological perturbation strategies, natural compounds are emphasized here because their frequently reported multi-target activities provide a testable context in which to examine whether perturbations can influence more than one ICA-associated regulatory dimension. This perturbational component is therefore presented as an application of the ICA-defined molecular state, rather than as a separate drug-discovery framework.

The first step constructs a directional ICA perturbation signature from differential transcriptional features between ICA-defined states. In the implemented workflow, differential-expression analysis between ICA-high and ICA-low tumors generates upregulated and downregulated query components, from which the most informative genes are used to construct a bidirectional perturbational signature. This signature is queried against compound-induced transcriptional profiles represented within the Connectivity Map (CMap)/Library of Integrated Network-Based Cellular Signatures (LINCS) framework. CMap established the principle that biological states and chemical perturbations can be related through concordant or opposing gene-expression signatures, while the L1000 platform substantially expanded perturbational profiling across chemical and genetic perturbations (Lamb et al., 2006; Subramanian et al., 2017). Compounds with transcriptional profiles inversely related to the ICA-associated query are consequently ranked as candidate perturbators for subsequent experimental evaluation.

A negative connectivity relationship is interpreted here as predicted transcriptional opposition to the queried ICA-associated state, rather than restoration of tumor immunogenicity or evidence of therapeutic activity. Perturbational relationships can vary according to cell lineage, dose, exposure duration, and molecular context; accordingly, candidate ranking should be interpreted together with the experimental context represented in the underlying perturbational profiles. Candidates supported only under isolated conditions should be distinguished from those exhibiting directionally consistent effects across multiple biologically compatible contexts. These connectivity relationships therefore serve as a hypothesis-generating prioritization layer and not as evidence of pharmacodynamic activity in cervical cancer.

The second step introduces network-level prioritization. Compound–target associations obtained from curated pharmacological resources or explicitly identified computational sources can be mapped onto the ICA regulatory network comprising epigenetic regulators, DDR components, and immune-signaling mediators. Network degree, proximity to ICA-associated modules, and cross-axis connectivity can then be used to identify compounds linked to multiple regulatory components. However, a network edge between a compound and an ICA-associated protein is not considered evidence of physical binding or functional target engagement unless supported by an appropriate experimental or curated pharmacological source. Network analysis is therefore used to refine candidate prioritization and generate mechanistic hypotheses rather than establish compound mechanism of action.

As a subsequent extension of perturbational prioritization, compound-induced transcriptional profiles can be rescored against the directional Epigenetic, DDR, and Immune gene sets used in ICA construction. Such analysis would provide an interpretable means of distinguishing perturbations predicted to predominantly affect one ICA dimension from those exhibiting coordinated multi-axis transcriptional effects. Candidate ranking could consequently integrate the magnitude and consistency of transcriptional opposition with independently supported compound–target relationships and cross-axis network connectivity. This extension provides a testable route toward multi-axis perturbation analysis but should not be interpreted as evidence that a prioritized compound functionally modulates all implicated ICA dimensions.

In the exploratory perturbational analysis, Figure 3 summarizes natural-compound candidates ordered by their compound-level connectivity scores, with more negative values indicating stronger transcriptional opposition to the ICA-associated query. Compound-associated network information is displayed alongside the ranking to indicate the number and distribution of ICA-related nodes linked to each candidate. No P-value threshold was applied. The analysis is intended solely to prioritize candidates for experimental testing and does not establish disease reversal, compound mechanism, target engagement, immunomodulatory activity, or therapeutic efficacy.

The resulting compound ranking is an exploratory step in the ICA analysis. Prioritized candidates require validation in independent laboratory assays, including confirmation of compound identity and purity, dose–response and cytotoxicity assessment, evaluation of proposed target engagement, measurement of relevant ICA component pathways, and functional assessment of antigen presentation, DNA-repair phenotypes, and immune-relevant responses. Where modulation of tumor immunogenicity is proposed, immune-competent systems, including appropriate co-culture models, will be necessary before immunomodulatory activity can be inferred. Thus, computational perturbation analysis narrows the experimental search space but neither establishes mechanism of action nor substitutes for biological validation.

### Disease model: cervical cancer

Cervical cancer provides a biologically coherent and clinically relevant system for evaluating the Immunogenicity Control Axis (ICA), as it represents a disease context in which viral oncogenesis, epigenetic reprogramming, DNA repair dysregulation, and immune evasion converge within a unified molecular landscape (Galluzzi et al., 2015; Vinay et al., 2015; The Cancer Genome Atlas Research Network, 2017). This positions cervical cancer as a model system for testing ICA-derived hypotheses and evaluating the proposed relationships among epigenetic, DDR, and immune-associated molecular states.

Human papillomavirus (HPV) oncoproteins, particularly E6 and E7, induce widespread epigenetic alterations that reshape chromatin architecture and transcriptional programs, including those governing antigen presentation and immune signaling pathways (Galluzzi et al., 2015; The Cancer Genome Atlas Research Network, 2017). Concurrently, HPV-mediated disruption of key tumor suppressor pathways interferes with DNA damage response mechanisms, leading to altered repair pathway utilization and increased genomic instability (Jeggo et al., 2016; The Cancer Genome Atlas Research Network, 2017). These upstream perturbations collectively influence both transcriptional accessibility and mutational landscapes, thereby directly impacting tumor immunogenicity.

Despite the presence of viral antigens that would be expected to promote immune recognition, cervical tumors frequently exhibit immune escape and limited responsiveness to immunotherapy (Galluzzi et al., 2015; Vinay et al., 2015). Within the ICA framework, this apparent paradox is explained by the presence of upstream regulatory constraints: epigenetic repression can limit antigen presentation despite antigen availability, while DDR pathway bias may generate suboptimal or non-immunogenic mutation profiles. This demonstrates that immune activation is not solely dependent on antigen presence but is contingent on coordinated regulation across the epigenetic and genomic layers.

Importantly, the ICA framework provides a mechanistic explanation for several clinically observed phenomena in cervical cancer. Variability in chemoradiation response can be interpreted through differences in DDR pathway activity and repair fidelity, which influence both treatment sensitivity and mutation-driven immunogenicity (Lord and Ashworth, 2017; Pilié et al., 2019; Kushwah et al., 2023). Similarly, inconsistent responses to immunotherapy may reflect heterogeneity in epigenetic accessibility and immune signaling engagement across tumors with otherwise comparable immune profiles (Sharma et al., 2017; Singh et al., 2025). The persistence of immune-cold phenotypes, despite viral oncogenesis, further underscores the role of upstream ICA constraints in limiting effective immune activation.

Collectively, these observations position cervical cancer as a model system in which the ICA can be both mechanistically interrogated and computationally validated. By integrating epigenetic, DDR, and immune features within a single framework, ICA enables a more comprehensive understanding of therapeutic variability and provides a foundation for stratification strategies and combination therapies tailored to tumor-specific regulatory states.

Cervical cancer therefore represents an appropriate initial disease context for ICA development because persistent HPV infection, extensive epigenetic remodeling, DNA repair dysregulation, and heterogeneous immune phenotypes converge within the same biological system.

## Testable hypotheses

To enable experimental and computational validation of the Immunogenicity Control Axis (ICA), we define the following testable hypotheses, each grounded in measurable molecular and phenotypic outputs:

H1 Epigenetic activation enhances antigen presentation capacity - Pharmacological or natural compound–mediated epigenetic modulation will increase chromatin accessibility at immune-relevant loci, leading to upregulation of MHC class I/II genes and antigen processing machinery. This can be quantified through transcriptomic profiling, chromatin accessibility proxies, and expression of antigen presentation markers (Seto and Yoshida, 2014; Roulois et al., 2015; Topper et al., 2017; Jones et al., 2019).

H2 DNA repair pathway bias determines neoantigen diversity and immunogenicity - Preferential engagement of error-prone DNA repair pathways, including non-homologous end joining (NHEJ) and alternative end joining (alt-EJ), will increase neoantigen diversity and alter mutational signatures. This can be assessed through genomic sequencing data and mutational signature analysis derived from large-scale datasets (Australian Pancreatic Cancer Genome Initiative et al., 2013; McGranahan et al., 2016).

H3 Coordinated modulation of epigenetic state and DDR enhances immune cytotoxicity - Simultaneous perturbation of epigenetic accessibility and DNA repair dynamics will produce synergistic increases in immune activation, reflected by elevated cytotoxic T-cell signatures, interferon signaling, and immune infiltration markers. These effects can be evaluated through integrated transcriptomic and immune profiling (Rooney et al., 2015; Ayers et al., 2017; Cristescu et al., 2018).

H4 Natural compounds induce coordinated perturbation of ICA components - Treatment with selected natural compounds will result in concurrent modulation of epigenetic, DDR, and immune pathways, detectable through multi-omics integration of gene expression, pathway activity, and immune signatures. This coordinated effect distinguishes ICA perturbation from single-layer modulation (Galluzzi et al., 2015; Jones et al., 2019).

H5 ICA-based stratification improves prediction of therapeutic response - The composite ICA Score will outperform individual biomarkers, such as PD-L1 expression or tumor mutation burden, in predicting response to immunotherapy and combination treatments. This can be validated through retrospective analysis of multi-omics datasets and clinical outcome correlations (Sharma et al., 2017; Cristescu et al., 2018; Thorsson et al., 2018).

Importantly, all hypotheses within the ICA framework are testable using existing multi-omics datasets, enabling immediate computational validation without dependence on *de novo* experimental systems.

Although cervical cancer provides a biologically informative setting in which to operationalize ICA because viral oncogenesis, epigenetic remodeling, DNA-repair dysregulation, and immune selection converge within the same disease context, the framework should not be assumed to transfer unchanged across tumor types. The relative informativeness of the Epigenetic, DDR, and Immune dimensions is likely to depend on tumor lineage, etiological context, genomic architecture, and the dominant mechanisms generating or restricting immune visibility. In tumors with low mutational burden, for example, mutation-derived antigenicity may contribute less to immunogenic variation, increasing the relative importance of epigenetic regulation, antigen-presentation competence, and other non-mutational determinants of immune visibility. Conversely, hematologic malignancies may require lineage-specific reformulation because their immune microenvironment, antigenic landscape, differentiation state, and recurrent genomic alterations differ fundamentally from those of epithelial solid tumors. Accordingly, extension of ICA beyond cervical cancer should preserve the three-dimensional biological logic while permitting tumor-specific feature selection, recalibration of component scores and integration weights, and independent validation of molecular and clinical associations, rather than applying the cervical-cancer implementation without modification.

### Validation roadmap

The purpose of the following validation roadmap is to define progressively stronger empirical tests of the central ICA hypothesis rather than to imply that its computational operationalizations already constitute biological or clinical validation. Validation of the Immunogenicity Control Axis (ICA) requires determining whether coordinated integration of epigenetic, DNA damage response (DDR), and immune features captures reproducible molecular information beyond that provided by the individual axes alone. Accordingly, the validation strategy is organized into three sequential levels: molecular reproducibility in independent multi-omics cohorts, systems-level interrogation of inter-axis relationships, and clinical benchmarking of ICA-derived models against established biomarkers. This staged design establishes an explicit progression from computational operationalization to biological evaluation and, ultimately, clinical validation, while defining the evidence required at each level to support or refute the ICA hypothesis.

## Multi-omics implementation and computational validation

Initial implementation of the ICA framework will use cervical cancer cohorts for which transcriptomic, genomic, epigenomic, and clinical annotations can be harmonized at the patient level. The Cancer Genome Atlas cervical squamous cell carcinoma and endocervical adenocarcinoma cohort (TCGA-CESC) provide an appropriate discovery resource, while independent Gene Expression Omnibus (GEO) or other publicly accessible cohorts may be used for replication only when the molecular features and relevant clinical endpoints required for a specified analysis are available (The Cancer Genome Atlas Research Network et al., 2013; Hoadley et al., 2014; The Cancer Genome Atlas Research Network, 2017; The Cancer Genome Atlas Research Network et al., 2013; Thorsson et al., 2018). Dataset eligibility, sample inclusion and exclusion criteria, endpoint definitions, missing-data handling, and molecular preprocessing should be prespecified before model evaluation.

For each eligible tumor, standardized Epigenetic, DDR, and Immune component scores will be calculated using the feature definitions and scoring strategy described in Section 5. Molecular state discovery will initially be performed without clinical outcome labels. The number and stability of ICA-associated states will be evaluated using consensus clustering, resampling-based stability measures, and internal cluster-validity indices. Associations with HPV status, FIGO stage, histological subtype, and other clinicopathological characteristics will subsequently be examined to determine whether the derived states capture molecular heterogeneity not explained by established disease classifications.

Reproducibility will be assessed at both the component-score and molecular-state levels. Where compatible independent datasets are available, the direction and magnitude of axis-level associations will be examined across cohorts, and state assignment will be evaluated using locked feature definitions or a prespecified nearest-centroid or classifier-based transfer strategy. Failure of a molecular state to reproduce across datasets will be interpreted as evidence requiring refinement of the corresponding ICA feature architecture rather than as confirmation of the proposed model. As an exploratory patient-derived analysis, we evaluated the association between the ICA Score and overall survival in TCGA-CESC. The 304 evaluable cases were dichotomized using the cohort median ICA Score, with values greater than or equal to the median classified as ICA-high (n = 152) and values below the median classified as ICA-low (n = 152). Kaplan–Meier analysis showed no statistically significant difference in overall survival between the ICA-high and ICA-low groups (HR = 1.19, 95% CI 0.90–1.57; log-rank P = 0.202; Figure 6). Thus, this analysis does not establish the ICA Score as a prognostic biomarker and is presented as exploratory in-cohort prognostic benchmarking requiring independent external validation.

**FIGURE 6:**
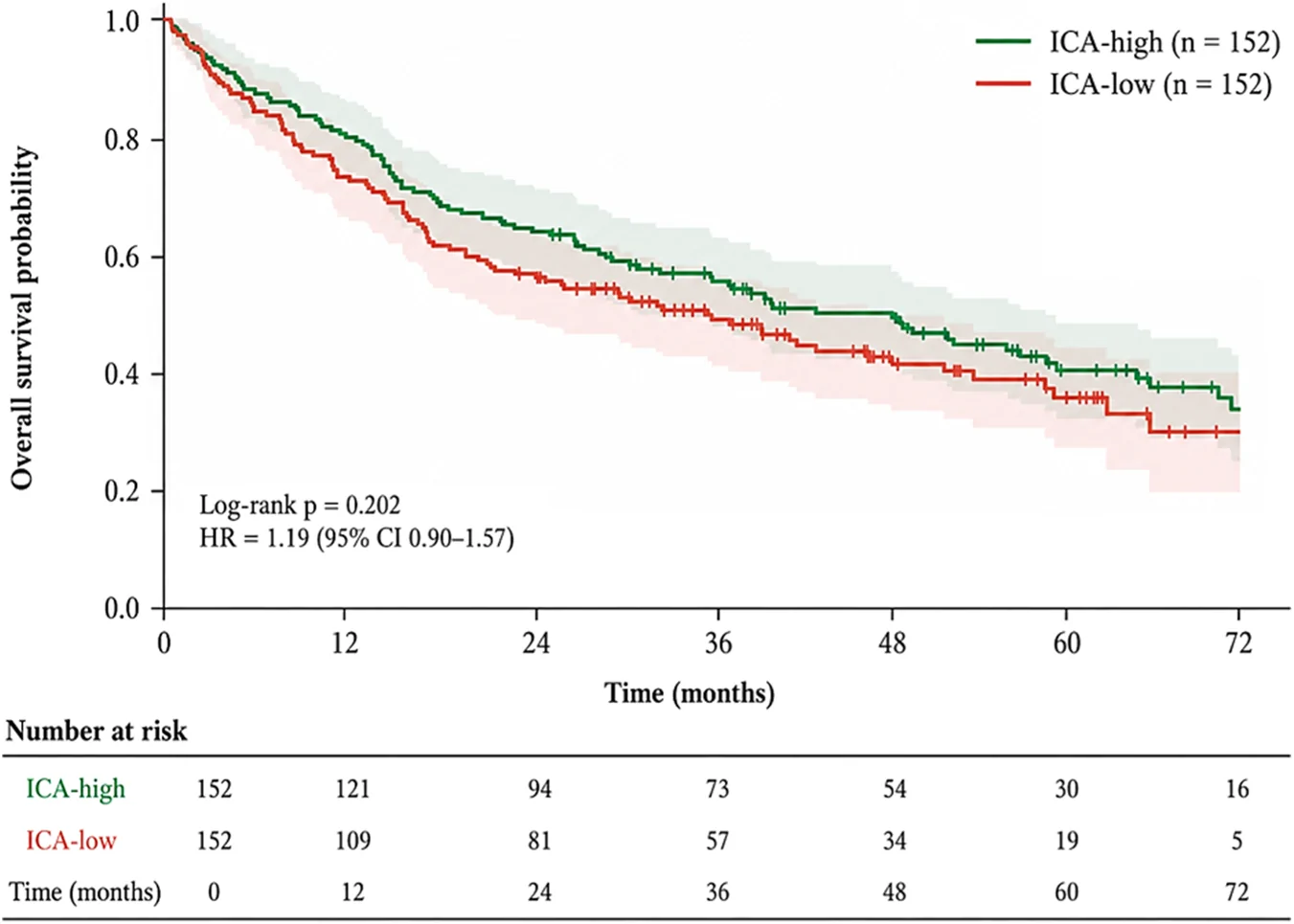
Exploratory association of the ICA Score with overall survival in TCGA-CESC. Kaplan–Meier analysis of overall survival among 304 evaluable TCGA-CESC cases dichotomized using the cohort median ICA Score. Cases with ICA Scores greater than or equal to the cohort median were classified as ICA-high (n = 152), whereas cases with scores below the median were classified as ICA-low (n = 152). Hazard ratios and 95% confidence intervals were estimated using Cox proportional-hazards regression, and survival distributions were compared using the log-rank test. No statistically significant difference in overall survival was observed between the groups (HR = 1.19, 95% CI 0.90–1.57; log-rank P = 0.202). This patient-derived analysis represents exploratory in-cohort prognostic benchmarking and not independent clinical validation of the ICA framework.

In patient-derived cohorts, prognostic evaluation will use consistently defined survival endpoints and censoring rules across all candidate biomarkers. Associations between the ICA Score and time-to-event outcomes will be estimated using Cox proportional-hazards models, with adjustment for available clinicopathological covariates including age, FIGO stage, histological subtype, and treatment exposure. Proportional-hazards assumptions will be formally assessed. Incremental prognostic information beyond PD-L1 and TMB will be evaluated using nested model comparison, changes in discrimination, and calibration rather than nominal log-rank significance alone. Where sample size permits, internal validation will use bootstrap resampling or nested cross-validation, followed by independent cohort validation using a locked scoring model.

Evaluation of treatment-response prediction will be analytically separated from prognosis. Predictive claims regarding immunotherapy or chemoradiation require cohorts with documented treatment exposure and standardized response endpoints. For such analyses, the ICA model will be tested for treatment-associated discrimination and, where an appropriate comparator group exists, treatment-by-ICA interaction. TCGA-CESC survival associations alone will not be interpreted as evidence of immunotherapy-response prediction.

### Systems-level interrogation of inter-axis relationships

Following molecular implementation, systems-level analyses will examine whether the three ICA axes exhibit reproducible regulatory relationships consistent with the proposed framework. Protein–protein interaction, transcriptional regulatory, and pathway-level networks will be constructed using prespecified ICA features and curated molecular interaction resources. Network centrality and community structure may be used to identify candidate convergence nodes connecting epigenetic regulation, DDR signaling, and immune-associated programs (Rooney et al., 2015; Ayers et al., 2017).

Computational perturbation and network-propagation approaches may then be used to estimate how alteration of individual nodes or axis-level modules changes the predicted state of the broader ICA network. These analyses are intended to prioritize directional relationships and experimentally testable nodes; they do not establish molecular causality or direct target engagement. Candidate relationships showing consistent cross-axis effects across analytical approaches will be prioritized for functional perturbation studies using genetically or pharmacologically defined experimental systems.

Natural compounds identified through the perturbation-signature workflow described in Section 5.1 will be evaluated within the same network architecture. Compound-associated transcriptional signatures and curated target information will be mapped onto ICA modules to quantify cross-axis connectivity and predicted state reversal. Network proximity or signature reversal will be treated as prioritization evidence only, with direct target engagement, dose dependence, and functional modulation requiring subsequent experimental validation.

The multilayer interaction network shown in Figure 7 illustrates this systems-level analytical strategy by integrating representative ICA-associated nodes with candidate compound–target relationships. Protein-associated interactions connect components of the Epigenetic, DDR, and Immune axes, while candidate compounds are mapped as a distinct interaction layer according to reported or computationally predicted target associations. The resulting topology summarizes inter-axis connectivity and compound–target intersections represented within the reconstructed network, providing a basis for prioritizing molecular nodes and candidate perturbators for subsequent analysis. Importantly, network connectivity is interpreted as hypothesis-generating evidence and does not establish causal regulatory direction, direct target engagement, or therapeutic efficacy.

**FIGURE 7:**
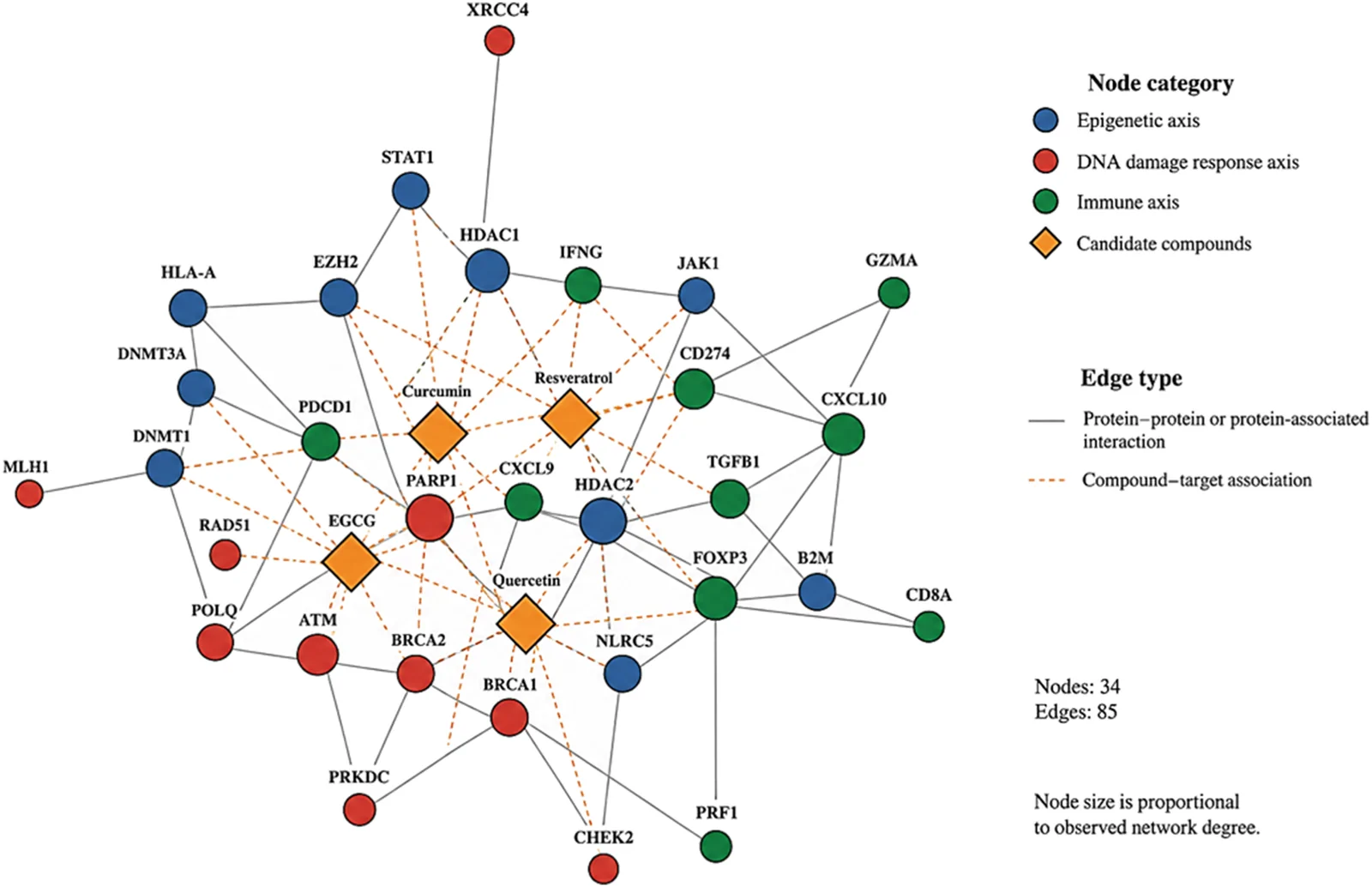
Multilayer interaction network linking Immunogenicity Control Axis regulatory nodes with candidate compound–target associations. The network integrates 30 regulatory genes assigned to the epigenetic, DNA damage response, and immune axes with four candidate compounds. Nodes are colored according to functional category, and node size is proportional to observed network degree. Solid grey edges represent within-axis or cross-axis protein-associated interactions included in the reconstructed network, whereas dashed orange edges represent compound–target associations incorporated through the network-analysis workflow. The reconstructed network contains 34 nodes and 85 edges, including 36 compound–target, 32 within-axis, and 17 cross-axis connections. The network is intended for hypothesis generation and candidate prioritization; network connectivity does not by itself establish physical target engagement, direct functional regulation, coordinated multi-axis modulation, or therapeutic efficacy.

### Translational benchmarking and model refinement

The translational objective of the ICA framework is to determine whether integration of epigenetic, DDR, and immune information provides clinically relevant information beyond established molecular and clinicopathological variables. ICA-derived models will therefore be benchmarked against PD-L1, TMB, individual ICA component scores, and available clinical covariates using identical analytical cohorts and endpoint definitions.

Model development will use prespecified training and validation procedures. Penalized regression or other interpretable multivariable approaches may be used to estimate ICA component weights when equal weighting is insufficient. Feature selection, weight estimation, and threshold determination will be restricted to training data and locked before validation. Performance will be assessed using endpoint-appropriate measures of discrimination and calibration, with decision-analytic assessment considered only after reproducible predictive performance has been demonstrated.

As an exploratory translational benchmark, receiver operating characteristic analysis was used to compare five models for classifying 3-year overall-survival status in the analyzed TCGA-CESC cohort. The models included the ICA composite model, an ICA-components model jointly incorporating the Epigenetic, DDR, and Immune Axis scores, a combined TMB + PD-L1 model, TMB alone, and PD-L1 expression alone. The ICA composite model showed the highest internally cross-validated discrimination (AUC = 0.931), followed by the ICA-components model (AUC = 0.923), the combined TMB + PD-L1 model (AUC = 0.890), TMB alone (AUC = 0.869), and PD-L1 expression alone (AUC = 0.823) (Figure 8). These AUCs represent internally cross-validated performance within TCGA-CESC and should not be interpreted as independently validated estimates of out-of-sample discrimination. The performance of the ICA-based models provides an exploratory rationale for further evaluation of multi-axis integration; however, its incremental prognostic value requires confirmation in independent cohorts and comparison with established clinicopathological models. These findings concern prognostic classification within TCGA-CESC and do not establish treatment-response or immunotherapy-predictive utility.

**FIGURE 8:**
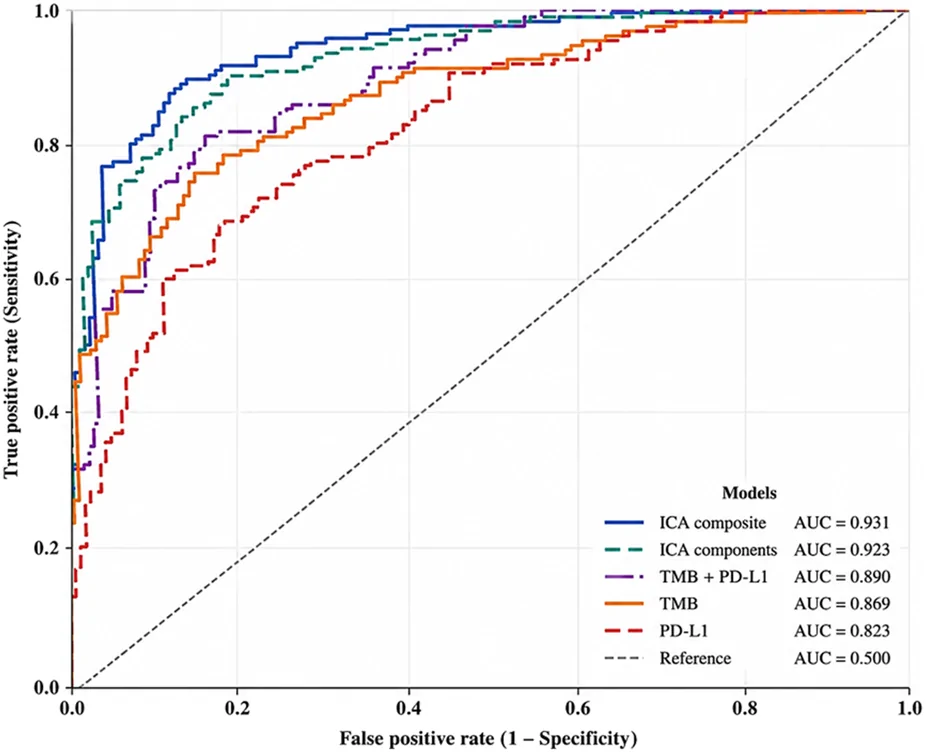
Internally cross-validated ROC analysis of ICA-based discrimination of 3-year overall-survival status in TCGA-CESC. ROC curves compare five models: the ICA composite model (AUC = 0.931); an ICA-components model jointly incorporating the Epigenetic, DDR, and Immune Axis scores (AUC = 0.923); a combined TMB + PD-L1 model (AUC = 0.890); TMB alone (AUC = 0.869); and PD-L1 expression alone (AUC = 0.823). AUCs were estimated using stratified five-fold cross-validation within the analyzed TCGA-CESC cohort and do not represent independent external validation. The grey diagonal represents chance-level discrimination (AUC = 0.500).

A clinically simplified ICA panel should be developed only if the full multi-omics model demonstrates reproducible incremental value. Candidate reduced panels may subsequently be derived using stability-based feature selection and evaluated for concordance with the full ICA Score. This sequential strategy prevents premature biomarker reduction before the underlying multi-axis framework has been validated.

Collectively, this roadmap establishes explicit criteria for progression of the ICA from an initial multi-omics model to a reproducibly validated translational framework. Evidence of reproducible axis-level structure would support molecular validity; reproducible inter-axis relationships would justify mechanistic investigation; and incremental performance in independently validated clinical cohorts would be required before prognostic or treatment-response claims are made. Failure at any stage would trigger refinement of feature definitions, weighting, or model architecture rather than retrospective reinterpretation of the validation criteria.

Several limitations should be considered during evaluation of the proposed framework. The current ICA model is primarily designed for implementation using retrospective multi-omics datasets and therefore requires prospective validation in independent clinical cohorts. The computational analyses proposed within the framework do not establish causal biological relationships and must be complemented by mechanistic experimental studies. In addition, the relative contribution of individual ICA components may vary across tumor types, reflecting differences in genomic, epigenetic, and immune landscapes. Finally, the performance of the ICA framework is expected to depend on the selection and standardization of pathway-specific features, highlighting the need for rigorous optimization and external validation before clinical application.

## Translational implications

The Immunogenicity Control Axis (ICA) provides a framework for linking multi-omics tumor stratification with mechanism-informed therapeutic prioritization. Rather than selecting combination strategies solely from isolated biomarker status or empirically observed synergy, ICA-defined molecular states could be used to identify regulatory constraints distributed across epigenetic, DNA damage response (DDR), and immune axes. This framework generates testable hypotheses for selecting interventions or combinations according to the dominant multi-axis configuration of an individual tumor.

The translational premise is that molecular constraints identified through ICA profiling may reveal rational points for therapeutic perturbation. For example, tumors characterized by restricted antigen-presentation-associated transcription together with an adverse immune state may warrant evaluation of epigenetic–immune combinations, whereas distinct DDR-associated configurations may motivate investigation of DNA repair-directed strategies in an appropriate genomic and immune context (Roulois et al., 2015; Lord and Ashworth, 2017; Sharma et al., 2017; Topper et al., 2017; Jones et al., 2019; Pilié et al., 2019). These relationships remain hypotheses for experimental and clinical testing and should not be interpreted as evidence that simultaneous multi-axis targeting will necessarily improve therapeutic response.

Candidate natural compounds provide one perturbational class through which this multi-axis hypothesis can be tested. Compounds prioritized through concordant transcriptional signature reversal and cross-axis network connectivity may be advanced to functional assays examining whether predicted ICA-state shifts occur under defined dose and exposure conditions. Their inclusion within the framework reflects their potential for multi-node perturbation rather than an assumption of clinical efficacy or superiority to established targeted agents (Galluzzi et al., 2015; Jones et al., 2019).

The internally cross-validated discrimination of the ICA composite and ICA-components models relative to the TMB- and PD-L1-based comparator models (Figure 8) provides an exploratory quantitative rationale for further evaluation of multi-axis biomarker strategies.

However, prognostic discrimination in TCGA-CESC does not establish treatment-response prediction. Translation of the ICA Score into therapeutic decision-making will require independent validation in treatment-annotated cohorts, assessment of incremental value beyond established clinicopathological variables, and prospective evaluation using prespecified analytical thresholds (Sharma et al., 2017; Cristescu et al., 2018; Thorsson et al., 2018).

## Conclusion

The Immunogenicity Control Axis (ICA) provides a testable framework for examining tumor immunogenicity through the coordinated assessment of epigenetic regulation, DNA damage response (DDR), and immune state. Its central proposition is that these dimensions should not be considered as isolated determinants of tumor immune visibility, but as biologically interacting processes whose joint evaluation may capture information not represented by individual molecular features alone. The ICA Score and associated molecular-state analyses provide quantitative operationalizations of this hypothesis rather than evidence that the underlying biological relationships are additive, causal, or clinically validated.

By combining multi-omics characterization with molecular stratification, outcome benchmarking, and perturbational analysis, ICA establishes a computationally tractable strategy for testing this hypothesis in patient-derived datasets and for prioritizing candidate perturbations for subsequent investigation. The cervical cancer implementation provides an exploratory proof of principle, while extension across tumor contexts will require context-specific calibration, independent replication, and experimental validation of the proposed inter-axis relationships. Accordingly, the principal value of ICA lies in providing a falsifiable framework that connects biological hypothesis with quantitative evaluation and defines a pathway from computational evidence to mechanistic and translational validation.

## Data Availability

All data generated or analyzed during this study are included in this published article and its Supplementary Material. No additional datasets were generated. All analysis scripts and computational codes used in this study are publicly available at https://github.com/Dr-SS03/ica-score-cervical-cancer-pipeline.
